# Supplementary figures and images for: Characterization of black patina from the Tiber River embankments using Next-Generation Sequencing
Source: PLoS One. 2020 Jan 9;15(1):e0227639. doi: 10.1371/journal.pone.0227639 (PMC6952188; doi:10.1371/journal.pone.0227639)

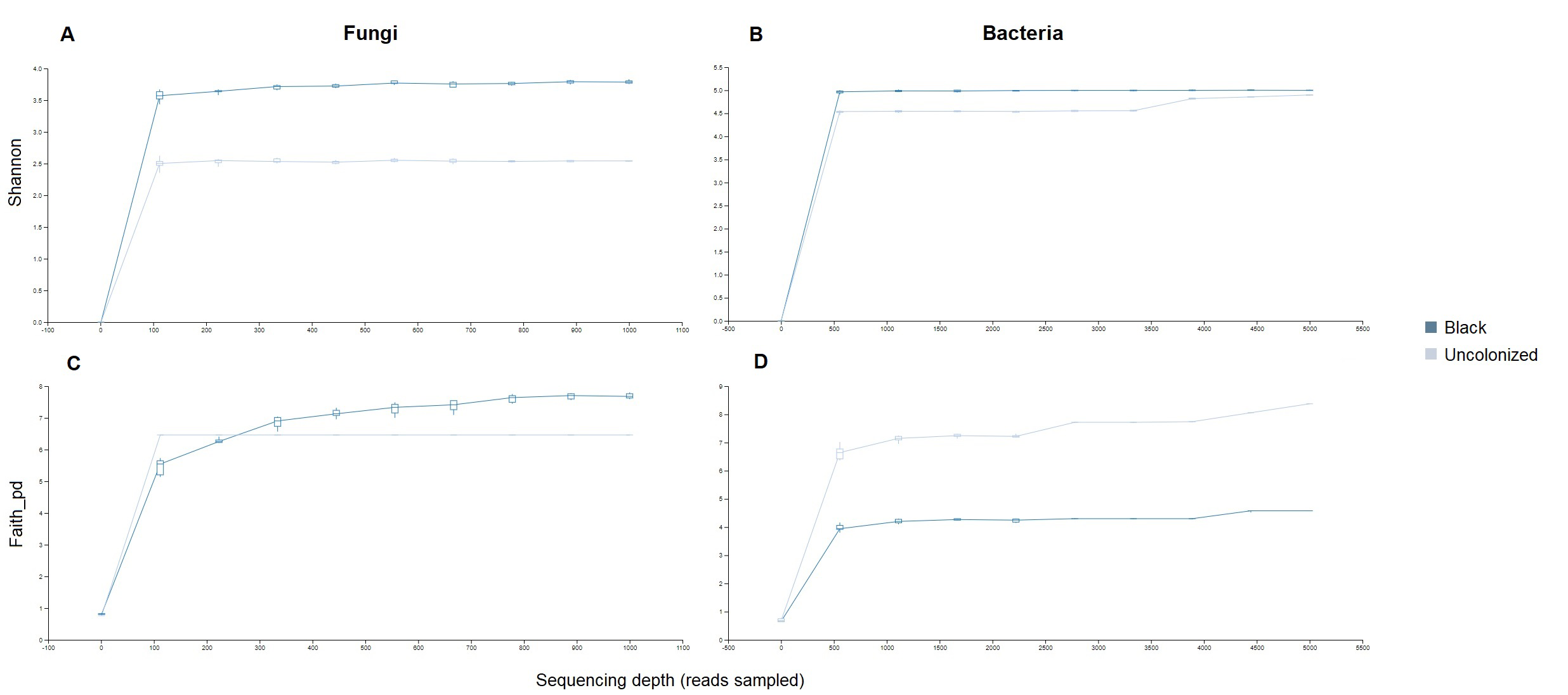

Supplement: S1 Fig — Rarefaction curves showing the Shannon values (A and B) and phylogenetic diversity (C and D) for ITS (A and C) and 16S rRNA (B and D). Values are grouped according to the type of samples (black patina vs. uncolonized controls) and displayed as box plots each with 500 reads. (TIF) [file pone.0227639.s001.tif]
