# Supplementary material for: Characterization of black patina from the Tiber River embankments using Next-Generation Sequencing
Source: PLoS One. 2020 Jan 9;15(1):e0227639. doi: 10.1371/journal.pone.0227639 (PMC6952188; doi:10.1371/journal.pone.0227639)
Supplement: S1 Table — Red color indicates a failed first quality check, whereas an asterisk (*) shows the samples excluded after rarefaction curve analysis. (DOCX) [file pone.0227639.s002.docx]

**Table S1**

**S1 Table. Metadata. Distinct sample IDs, specific amount of DNA extraction and corresponding obtained library.** Red color indicates a failed first quality check, whereas “*” symbol shows the samples excluded after rarefaction curve analysis.

| **Sample ID** | **DNA extraction** | **Library prep 16S** | **Library prep ITS2** | **Library Nextera** |
| --- | --- | --- | --- | --- |
| U1 | 0.3 ng/ul |  |  |  |
| U2 | 0.7 ng/ul |  |  |  |
| U3 | 0 |  |  |  |
| U4 | 0 |  |  |  |
| U5 | 0.3 ng/ul | **X** | **X*** |  |
| U6 | 0.5 ng/ul | **X** |  |  |
| U7 | 0.5 ng/ul | **X** |  |  |
| U8 | 4.1 ng/ul | **X** | **X*** | X |
| U9 | 0.6 ng/ul | **X** | **X** |  |
| U10 | 0.7 ng/ul | **X** | **X*** |  |
| U11 | 1.7 ng/ul | X |  |  |
| U12 | 0.3 ng/ul |  |  |  |
| U13 | 0.3 ng/ul |  |  |  |
| U14 | 0 |  |  |  |
| U15 | 1.8 ng/ul | X |  |  |
| U16 | 2.3 ng/ul | **X** |  | X |
| U17 | 0 |  |  |  |
| U18 | 1.2 ng/ul | **X** |  |  |
| U19 | 2.5 ng/ul | **X** | **X*** | X |
| U20 | 2.3 ng/ul | **X*** | **X*** |  |
| B1 | 13.3 ng/ul | **X** | **X** | X |
| B2 | 11.5 ng/ul | **X** | **X** | X |
| B3 | 34.2 ng/ul | **X** | **X** | X |
| B4 | 11.2 ng/ul | **X** | **X** | X |
| B5 | 13.9 ng/ul | **X** | **X** | X |
| B6 | 11.6 ng/ul | **X** | **X** | X |
| B7 | 8.1 ng/ul | **X** | **X** | X |
| B8 | 8.2 ng/ul | **X** | **X** | X |
| B9 | 12.8 ng/ul | **X** | **X** | X |
| B10 | 19.2 ng/ul | **X** | **X** | X |
| B11 | 14.2 ng/ul | **X** | **X** | X |
| B12 | 12.9 ng/ul | **X** | **X** | X |
| B13 | 9.7 ng/ul | **X** | **X** | X |
| B14 | 9 ng/ul | **X** | **X** |  |
| B15 | 10.1 ng/ul | **X** | **X** |  |
| B16 | 8.5 ng/ul | **X** | **X** |  |
| B17 | 7.2 ng/ul | **X** | **X** |  |
| B18 | 10 ng/ul | **X** | **X** |  |
| B19 | 7.8 ng/ul | **X** | **X** |  |
| B20 | 7.7 ng/ul | **X** | **X** |  |
