# Supplementary material for: Characterization of black patina from the Tiber River embankments using Next-Generation Sequencing
Source: PLoS One. 2020 Jan 9;15(1):e0227639. doi: 10.1371/journal.pone.0227639 (PMC6952188; doi:10.1371/journal.pone.0227639)
Supplement: S2 Table — (DOCX) [file pone.0227639.s003.docx]

**Table S2**

**S2 Table. Average taxonomic abundance (with standard deviation) for each bacterial family in uncolonized controls and Black patina samples.**

| **OTUs taxonomic assignment** | **Uncolonized** | **sd** | **Black patina** | **sd** |
| --- | --- | --- | --- | --- |
| Acidobacteria;D_2__Acidobacteriia;D_3__Solibacterales;D_4__Solibacteraceae (Subgroup 3) | 0.00 | 0.00 | 0.04 | 0.16 |
| Acidobacteria;D_2__Blastocatellia (Subgroup 4);D_3__Blastocatellales;D_4__Blastocatellaceae | 0.00 | 0.00 | 9.56 | 2.59 |
| Acidobacteria;D_2__Blastocatellia (Subgroup 4);D_3__Pyrinomonadales;D_4__Pyrinomonadaceae | 0.00 | 0.00 | 0.17 | 0.40 |
| Acidobacteria;D_2__Blastocatellia (Subgroup 4);__;__ | 0.00 | 0.00 | 0.82 | 0.50 |
| Actinobacteria;D_2__Acidimicrobiia;D_3__Microtrichales;D_4__Ilumatobacteraceae | 0.00 | 0.00 | 0.01 | 0.03 |
| Actinobacteria;D_2__Actinobacteria;D_3__Bifidobacteriales;D_4__Bifidobacteriaceae | 0.06 | 0.11 | 0.00 | 0.00 |
| Actinobacteria;D_2__Actinobacteria;D_3__Frankiales;D_4__Geodermatophilaceae | 0.00 | 0.00 | 0.23 | 0.62 |
| Actinobacteria;D_2__Actinobacteria;D_3__Frankiales;D_4__uncultured | 0.00 | 0.00 | 0.21 | 0.58 |
| Actinobacteria;D_2__Actinobacteria;D_3__Frankiales;__ | 0.00 | 0.00 | 0.12 | 0.47 |
| Actinobacteria;D_2__Actinobacteria;D_3__Kineosporiales;D_4__Kineosporiaceae | 0.00 | 0.00 | 2.49 | 1.75 |
| Actinobacteria;D_2__Actinobacteria;D_3__Micrococcales;D_4__Microbacteriaceae | 0.00 | 0.00 | 0.02 | 0.08 |
| Actinobacteria;D_2__Actinobacteria;D_3__Propionibacteriales;D_4__Propionibacteriaceae | 0.00 | 0.00 | 0.11 | 0.18 |
| Actinobacteria;D_2__Actinobacteria;D_3__Pseudonocardiales;D_4__Pseudonocardiaceae | 0.00 | 0.00 | 0.27 | 0.82 |
| Actinobacteria;D_2__Coriobacteriia;D_3__Coriobacteriales;D_4__Eggerthellaceae | 0.02 | 0.06 | 0.00 | 0.00 |
| Actinobacteria;D_2__Rubrobacteria;D_3__Rubrobacterales;D_4__Rubrobacteriaceae | 0.00 | 0.00 | 0.21 | 0.23 |
| Actinobacteria;D_2__Thermoleophilia;D_3__Solirubrobacterales;D_4__Solirubrobacteraceae | 0.00 | 0.00 | 0.02 | 0.08 |
| Armatimonadetes;D_2__Armatimonadia;D_3__Armatimonadales;D_4__uncultured bacterium | 0.00 | 0.00 | 0.56 | 0.55 |
| Armatimonadetes;D_2__Armatimonadia;D_3__Armatimonadales;__ | 0.00 | 0.00 | 0.06 | 0.23 |
| Armatimonadetes;D_2__Fimbriimonadia;D_3__Fimbriimonadales;D_4__Fimbriimonadaceae | 0.00 | 0.00 | 0.07 | 0.16 |
| Bacteroidetes;D_2__Bacteroidia;D_3__Bacteroidales;D_4__Bacteroidaceae | 2.50 | 0.92 | 0.00 | 0.00 |
| Bacteroidetes;D_2__Bacteroidia;D_3__Bacteroidales;D_4__Barnesiellaceae | 0.03 | 0.10 | 0.00 | 0.00 |
| Bacteroidetes;D_2__Bacteroidia;D_3__Bacteroidales;D_4__Marinifilaceae | 0.67 | 0.52 | 0.00 | 0.00 |
| Bacteroidetes;D_2__Bacteroidia;D_3__Bacteroidales;D_4__Muribaculaceae | 23.82 | 6.23 | 0.01 | 0.05 |
| Bacteroidetes;D_2__Bacteroidia;D_3__Bacteroidales;D_4__Prevotellaceae | 9.40 | 2.48 | 0.00 | 0.00 |
| Bacteroidetes;D_2__Bacteroidia;D_3__Bacteroidales;D_4__Rikenellaceae | 0.34 | 0.51 | 0.00 | 0.00 |
| Bacteroidetes;D_2__Bacteroidia;D_3__Bacteroidales;D_4__Tannerellaceae | 0.60 | 0.59 | 0.00 | 0.00 |
| Bacteroidetes;D_2__Bacteroidia;D_3__Bacteroidales;__ | 6.17 | 1.22 | 0.01 | 0.04 |
| Bacteroidetes;D_2__Bacteroidia;D_3__Chitinophagales;D_4__Chitinophagaceae | 0.00 | 0.00 | 5.93 | 1.06 |
| Bacteroidetes;D_2__Bacteroidia;D_3__Chitinophagales;__ | 0.00 | 0.00 | 0.06 | 0.17 |
| Bacteroidetes;D_2__Bacteroidia;D_3__Cytophagales;D_4__Cytophagaceae | 0.00 | 0.00 | 0.40 | 0.28 |
| Bacteroidetes;D_2__Bacteroidia;D_3__Cytophagales;D_4__Hymenobacteraceae | 0.00 | 0.00 | 1.99 | 1.31 |
| Bacteroidetes;D_2__Bacteroidia;D_3__Cytophagales;D_4__Spirosomaceae | 0.00 | 0.00 | 9.45 | 2.16 |
| Bacteroidetes;D_2__Bacteroidia;D_3__Cytophagales;__ | 0.00 | 0.00 | 0.48 | 0.51 |
| Bacteroidetes;D_2__Bacteroidia;D_3__Flavobacteriales;D_4__Weeksellaceae | 0.00 | 0.00 | 0.01 | 0.03 |
| Bacteroidetes;D_2__Bacteroidia;D_3__Sphingobacteriales;D_4__env.OPS 17 | 0.00 | 0.00 | 0.07 | 0.07 |
| Bacteroidetes;D_2__Bacteroidia;__;__ | 0.37 | 0.51 | 0.01 | 0.05 |
| Bacteroidetes;D_2__Rhodothermia;D_3__Rhodothermales;D_4__Rhodothermaceae | 0.00 | 0.00 | 0.01 | 0.03 |
| Chloroflexi;D_2__Chloroflexia;D_3__Chloroflexales;D_4__Herpetosiphonaceae | 0.00 | 0.00 | 0.01 | 0.03 |
| Chloroflexi;D_2__Chloroflexia;D_3__Kallotenuales;D_4__AKIW781 | 0.00 | 0.00 | 1.85 | 0.51 |
| Cyanobacteria;D_2__Melainabacteria;D_3__Gastranaerophilales;D_4__uncultured bacterium | 0.06 | 0.16 | 0.00 | 0.00 |
| Cyanobacteria;D_2__Oxyphotobacteria;D_3__Chloroplast;__ | 0.09 | 0.19 | 0.17 | 0.37 |
| Cyanobacteria;D_2__Oxyphotobacteria;D_3__Nostocales;D_4__Chroococcidiopsaceae | 0.00 | 0.00 | 29.02 | 4.87 |
| Cyanobacteria;D_2__Oxyphotobacteria;D_3__Nostocales;D_4__Nostocaceae | 0.00 | 0.00 | 0.56 | 2.17 |
| Cyanobacteria;D_2__Oxyphotobacteria;D_3__Nostocales;D_4__uncultured | 0.00 | 0.00 | 1.67 | 1.49 |
| Cyanobacteria;D_2__Oxyphotobacteria;D_3__Nostocales;__ | 0.00 | 0.00 | 1.56 | 1.40 |
| Deferribacteres;D_2__Deferribacteres;D_3__Deferribacterales;D_4__Deferribacteraceae | 0.03 | 0.10 | 0.00 | 0.00 |
| Deinococcus-Thermus;D_2__Deinococci;D_3__Deinococcales;D_4__Deinococcaceae | 0.00 | 0.00 | 0.22 | 0.39 |
| Deinococcus-Thermus;D_2__Deinococci;D_3__Deinococcales;D_4__Trueperaceae | 0.00 | 0.00 | 1.52 | 0.39 |
| Epsilonbacteraeota;D_2__Campylobacteria;D_3__Campylobacterales;D_4__Helicobacteraceae | 5.70 | 2.74 | 0.00 | 0.00 |
| FBP;D_2__uncultured bacterium;D_3__uncultured bacterium;D_4__uncultured bacterium | 0.00 | 0.00 | 1.87 | 0.67 |
| FBP;__;__;__ | 0.00 | 0.00 | 0.20 | 0.31 |
| Firmicutes;D_2__Bacilli;D_3__Lactobacillales;D_4__Lactobacillaceae | 0.07 | 0.16 | 0.00 | 0.00 |
| Firmicutes;D_2__Bacilli;D_3__Lactobacillales;D_4__Leuconostocaceae | 0.06 | 0.19 | 0.00 | 0.00 |
| Firmicutes;D_2__Clostridia;D_3__Clostridiales;D_4__Christensenellaceae | 0.02 | 0.06 | 0.00 | 0.00 |
| Firmicutes;D_2__Clostridia;D_3__Clostridiales;D_4__Clostridiaceae 1 | 0.14 | 0.44 | 0.00 | 0.00 |
| Firmicutes;D_2__Clostridia;D_3__Clostridiales;D_4__Clostridiales vadinBB60 group | 0.17 | 0.28 | 0.00 | 0.00 |
| Firmicutes;D_2__Clostridia;D_3__Clostridiales;D_4__Family XIII | 0.02 | 0.06 | 0.00 | 0.00 |
| Firmicutes;D_2__Clostridia;D_3__Clostridiales;D_4__Lachnospiraceae | 26.12 | 4.71 | 0.01 | 0.03 |
| Firmicutes;D_2__Clostridia;D_3__Clostridiales;D_4__Peptostreptococcaceae | 0.03 | 0.10 | 0.00 | 0.00 |
| Firmicutes;D_2__Clostridia;D_3__Clostridiales;D_4__Ruminococcaceae | 13.28 | 2.08 | 0.00 | 0.00 |
| Firmicutes;D_2__Clostridia;D_3__Clostridiales;__ | 0.37 | 0.63 | 0.00 | 0.00 |
| Firmicutes;D_2__Clostridia;__;__ | 0.09 | 0.29 | 0.00 | 0.00 |
| Firmicutes;D_2__Erysipelotrichia;D_3__Erysipelotrichales;D_4__Erysipelotrichaceae | 0.15 | 0.21 | 0.00 | 0.00 |
| Firmicutes;__;__;__ | 0.46 | 0.51 | 0.00 | 0.00 |
| Gemmatimonadetes;D_2__Gemmatimonadetes;D_3__Gemmatimonadales;D_4__Gemmatimonadaceae | 0.00 | 0.00 | 1.31 | 0.52 |
| Patescibacteria;D_2__Saccharimonadia;D_3__Saccharimonadales;__ | 0.00 | 0.00 | 0.21 | 0.28 |
| Planctomycetes;D_2__Phycisphaerae;D_3__Tepidisphaerales;D_4__Tepidisphaeraceae | 0.00 | 0.00 | 0.01 | 0.03 |
| Planctomycetes;D_2__Phycisphaerae;D_3__Tepidisphaerales;D_4__WD2101 soil group | 0.00 | 0.00 | 0.01 | 0.05 |
| Proteobacteria;D_2__Alphaproteobacteria;D_3__Acetobacterales;D_4__Acetobacteraceae | 0.00 | 0.00 | 2.30 | 0.73 |
| Proteobacteria;D_2__Alphaproteobacteria;D_3__Caedibacterales;D_4__Caedibacteraceae | 0.00 | 0.00 | 0.05 | 0.08 |
| Proteobacteria;D_2__Alphaproteobacteria;D_3__Caulobacterales;D_4__Caulobacteraceae | 0.00 | 0.00 | 0.39 | 0.35 |
| Proteobacteria;D_2__Alphaproteobacteria;D_3__Rhizobiales;D_4__Beijerinckiaceae | 0.00 | 0.00 | 0.14 | 0.39 |
| Proteobacteria;D_2__Alphaproteobacteria;D_3__Rhizobiales;D_4__Devosiaceae | 0.00 | 0.00 | 0.19 | 0.49 |
| Proteobacteria;D_2__Alphaproteobacteria;D_3__Rhizobiales;__ | 0.00 | 0.00 | 0.82 | 0.51 |
| Proteobacteria;D_2__Alphaproteobacteria;D_3__Rhodobacterales;D_4__Rhodobacteraceae | 0.00 | 0.00 | 7.20 | 1.33 |
| Proteobacteria;D_2__Alphaproteobacteria;D_3__Rhodospirillales;D_4__uncultured | 0.03 | 0.10 | 0.00 | 0.00 |
| Proteobacteria;D_2__Alphaproteobacteria;D_3__Sphingomonadales;D_4__Sphingomonadaceae | 0.00 | 0.00 | 14.46 | 2.90 |
| Proteobacteria;D_2__Alphaproteobacteria;__;__ | 0.00 | 0.00 | 0.05 | 0.11 |
| Proteobacteria;D_2__Deltaproteobacteria;D_3__Desulfovibrionales;D_4__Desulfovibrionaceae | 0.03 | 0.10 | 0.00 | 0.00 |
| Proteobacteria;D_2__Gammaproteobacteria;D_3__Betaproteobacteriales;D_4__Burkholderiaceae | 0.00 | 0.00 | 0.10 | 0.19 |
| Proteobacteria;D_2__Gammaproteobacteria;D_3__Pseudomonadales;D_4__Moraxellaceae | 0.01 | 0.03 | 0.00 | 0.00 |
| Proteobacteria;D_2__Gammaproteobacteria;D_3__Xanthomonadales;D_4__Xanthomonadaceae | 0.00 | 0.00 | 0.05 | 0.11 |
| Tenericutes;D_2__Mollicutes;D_3__Anaeroplasmatales;D_4__Anaeroplasmataceae | 0.04 | 0.08 | 0.00 | 0.00 |
| Tenericutes;D_2__Mollicutes;D_3__Mycoplasmatales;D_4__Mycoplasmataceae | 0.02 | 0.06 | 0.00 | 0.00 |
| Verrucomicrobia;D_2__Verrucomicrobiae;D_3__Verrucomicrobiales;D_4__Akkermansiaceae | 5.83 | 3.59 | 0.00 | 0.00 |
| D_0__Bacteria;__;__;__;__ | 0.42 | 0.52 | 0.58 | 0.50 |
| Unassigned;__;__;__;__ | 2.83 | 1.60 | 0.09 | 0.15 |
