# Supplementary material for: Characterization of black patina from the Tiber River embankments using Next-Generation Sequencing
Source: PLoS One. 2020 Jan 9;15(1):e0227639. doi: 10.1371/journal.pone.0227639 (PMC6952188; doi:10.1371/journal.pone.0227639)
Supplement: S3 Table — (DOCX) [file pone.0227639.s004.docx]

**Table S3**

**S3 Table. Taxonomic assignment inferred by blast on the NCBI 16S ribosomal sequence database of unclassified reads at the level of phylum (unclassified Cyanobacteria) and kingdom (unclassified Bacteria).**

|  | **Best Hit** | **Score** | **Query cov** | **E-val** | **perc. ID** | **acc.no.** |
| --- | --- | --- | --- | --- | --- | --- |
| **Unclassified Cyanobacteria** | Aliterella antarctica strain CENA408 16S ribosomal RNA, partial sequence | 411 | 100% | 2.00E-114 | 97.14% | NR_151904.1 |
|  | Aliterella antarctica strain CENA408 16S ribosomal RNA, partial sequence | 416 | 100% | 4.00E-116 | 97.55% | NR_151904.1 |
|  | Anabaena cylindrica PCC 7122 16S ribosomal RNA, partial sequence | 312 | 98% | 1.00E-84 | 88.84% | NR_102457.1 |
|  | Brasilonema octagenarum strain UFV-E1 16S ribosomal RNA, partial sequence | 399 | 98% | 3.00E-111 | 96.71% | NR_115956.1 |
|  | Calochaete cimrmanii strain RKST551 16S ribosomal RNA, partial sequence | 293 | 93% | 3.00E-79 | 88.65% | NR_117712.2 |
|  | Chamaesiphon minutus strain PCC 6605 16S ribosomal RNA, partial sequence | 368 | 90% | 2.00E-101 | 96.83% | NR_102459.1 |
|  | Chamaesiphon minutus strain PCC 6605 16S ribosomal RNA, partial sequence | 356 | 89% | 3.00E-98 | 95.93% | NR_102459.1 |
|  | Chamaesiphon minutus strain PCC 6605 16S ribosomal RNA, partial sequence | 368 | 90% | 2.00E-101 | 96.83% | NR_102459.1 |
|  | Chamaesiphon minutus strain PCC 6605 16S ribosomal RNA, partial sequence | 361 | 89% | 3.00E-99 | 96.38% | NR_102459.1 |
|  | Chamaesiphon minutus strain PCC 6605 16S ribosomal RNA, partial sequence | 372 | 90% | 4.00E-103 | 97.29% | NR_102459.1 |
|  | Chlorogloeopsis fritschii PCC 6912 16S ribosomal RNA, partial sequence | 294 | 89% | 3.00E-79 | 89.55% | NR_112176.1 |
|  | Chlorogloeopsis fritschii PCC 6912 16S ribosomal RNA, partial sequence | 289 | 89% | 4.00E-78 | 89.09% | NR_112176.1 |
|  | Chroococcidiopsis thermalis PCC 7203 16S ribosomal RNA, partial sequence | 419 | 99% | 3.00E-117 | 98.36% | NR_102464.1 |
|  | Cylindrospermum licheniforme strain CCALA 995 16S ribosomal RNA, partial sequence | 393 | 93% | 4.00E-109 | 97.83% | NR_125686.1 |
|  | Fischerella muscicola PCC 7414 16S ribosomal RNA, partial sequence | 288 | 86% | 1.00E-77 | 90.52% | NR_112107.1 |
|  | Hassallia antarctica strain CCALA 957 16S ribosomal RNA, partial sequence | 383 | 86% | 2.00E-106 | 100.00% | NR_117192.1 |
|  | Kastovskya adunca strain ATA6-11-RM4 16S ribosomal RNA, partial sequence | 398 | 93% | 1.00E-110 | 98.26% | NR_125700.1 |
|  | Kastovskya adunca strain ATA6-11-RM4 16S ribosomal RNA, partial sequence | 393 | 93% | 4.00E-109 | 97.83% | NR_125700.1 |
|  | Kryptousia macronema strain CENA338 16S ribosomal RNA, partial sequence | 387 | 98% | 2.00E-107 | 95.47% | NR_157980.1 |
|  | Kryptousia macronema strain CENA338 16S ribosomal RNA, partial sequence | 327 | 98% | 1.00E-89 | 90.04% | NR_157980.1 |
|  | Kryptousia microlepis strain CENA354 16S ribosomal RNA, partial sequence | 397 | 94% | 3.00E-110 | 97.84% | NR_157979.1 |
|  | Kryptousia microlepis strain CENA354 16S ribosomal RNA, partial sequence | 392 | 94% | 4.00E-109 | 97.41% | NR_157979.1 |
|  | Kryptousia microlepis strain CENA354 16S ribosomal RNA, partial sequence | 396 | 98% | 3.00E-110 | 96.71% | NR_157979.1 |
|  | Kryptousia microlepis strain CENA354 16S ribosomal RNA, partial sequence | 392 | 94% | 4.00E-109 | 97.41% | NR_157979.1 |
|  | Kryptousia microlepis strain CENA354 16S ribosomal RNA, partial sequence | 401 | 94% | 8.00E-112 | 98.28% | NR_157979.1 |
|  | Nostoc punctiforme PCC 73102 16S ribosomal RNA, complete sequence | 412 | 99% | 5.00E-115 | 97.53% | NR_074317.1 |
|  | Nostoc punctiforme PCC 73102 16S ribosomal RNA, complete sequence | 416 | 99% | 4.00E-116 | 97.94% | NR_074317.1 |
|  | Nostoc punctiforme PCC 73102 16S ribosomal RNA, complete sequence | 421 | 99% | 9.00E-118 | 98.35% | NR_074317.1 |
|  | Sinosporangium siamense strain A-T 1946 16S ribosomal RNA, partial sequence | 279 | 91% | 7.00E-75 | 88.44% | NR_134175.1 |
|  | Vampirovibrio chlorellavorus strain ICPB 3707 16S ribosomal RNA, partial sequence | 265 | 98% | 1.00E-70 | 84.77% | NR_104911.1 |
| **Unclassified Bacteria** | Deinococcus aetherius strain ST0316 16S ribosomal RNA, partial sequence | 389 | 100% | 5.00E-108 | 95.10% | NR_112189.1 |
|  | Parabacteroides merdae strain JCM 9497 16S ribosomal RNA, partial sequence | 328 | 95% | 1.00E-89 | 91.03% | NR_041343.1 |
|  | Sphingobacterium chuzhouense strain DH-5 16S ribosomal RNA, partial sequence | 230 | 97% | 3.00E-60 | 81.74% | NR_153692.1 |
|  | Truepera radiovictrix DSM 17093 strain RQ-24 16S ribosomal RNA, partial sequence | 383 | 97% | 2.00E-106 | 95.82% | NR_074381.1 |
|  | [Clostridium] viride strain T2-7 16S ribosomal RNA, partial sequence | 269 | 97% | 1.00E-71 | 84.71% | NR_026204.1 |
|  | Paludibaculum fermentans strain P105 16S ribosomal RNA, partial sequence | 324 | 100% | 2.00E-88 | 89.84% | NR_134120.1 |
|  | Brevitalea deliciosa strain Ac_16_C4 16S ribosomal RNA, partial sequence | 314 | 98% | 3.00E-85 | 88.80% | NR_151988.1 |
|  | Paludibaculum fermentans strain P105 16S ribosomal RNA, partial sequence | 320 | 100% | 2.00E-87 | 89.43% | NR_134120.1 |
|  | Brevitalea deliciosa strain Ac_16_C4 16S ribosomal RNA, partial sequence | 320 | 98% | 2.00E-87 | 89.26% | NR_151988.1 |
|  | Tepidisphaera mucosa strain 2842 16S ribosomal RNA, partial sequence | 393 | 100% | 4.00E-109 | 95.51% | NR_148582.1 |
|  | [Clostridium] viride strain T2-7 16S ribosomal RNA, partial sequence | 269 | 97% | 1.00E-71 | 84.71% | NR_026204.1 |
|  | Armatimonas rosea strain YO-36 16S ribosomal RNA, partial sequence | 237 | 89% | 2.00E-62 | 84.55% | NR_113009.1 |
|  | Deinococcus aetherius strain ST0316 16S ribosomal RNA, partial sequence | 391 | 99% | 1.00E-108 | 95.49% | NR_112189.1 |
|  | Paludibaculum fermentans strain P105 16S ribosomal RNA, partial sequence | 315 | 100% | 9.00E-86 | 89.02% | NR_134120.1 |
|  | Deinococcus aetherius strain ST0316 16S ribosomal RNA, partial sequence | 393 | 100% | 4.00E-109 | 95.51% | NR_112189.1 |
|  | Christensenella minuta strain YIT 12065 16S ribosomal RNA, partial sequence | 299 | 97% | 7.00E-81 | 87.60% | NR_112900.1 |
|  | Christensenella massiliensis strain Marseille-P2438 16S ribosomal RNA, partial sequence | 308 | 99% | 1.00E-83 | 88.07% | NR_144742.1 |
|  | Truepera radiovictrix DSM 17093 strain RQ-24 16S ribosomal RNA, partial sequence | 379 | 97% | 9.00E-105 | 95.40% | NR_074381.1 |
|  | Tepidisphaera mucosa strain 2842 16S ribosomal RNA, partial sequence | 301 | 100% | 2.00E-81 | 87.40% | NR_148582.1 |
|  | Terribacillus halophilus strain 002-051 16S ribosomal RNA, partial sequence | 291 | 98% | 1.00E-78 | 87.30% | NR_041357.1 |
|  | Christensenella massiliensis strain Marseille-P2438 16S ribosomal RNA, partial sequence | 304 | 99% | 2.00E-82 | 87.65% | NR_144742.1 |
|  | Paludibaculum fermentans strain P105 16S ribosomal RNA, partial sequence | 315 | 100% | 9.00E-86 | 89.02% | NR_134120.1 |
|  | No Hits | | | | | |
|  | Dongia soli strain D78 16S ribosomal RNA, partial sequence | 278 | 100% | 2.00E-74 | 85.31% | NR_146690.1 |
|  | Helicobacter muridarum strain ST1 16S ribosomal RNA, partial sequence | 280 | 98% | 2.00E-75 | 86.01% | NR_025939.1 |
|  | Butyricicoccus faecihominis strain KS-2 16S ribosomal RNA, partial sequence | 315 | 99% | 9.00E-86 | 88.48% | NR_152060.1 |
|  | Phocea massiliensis strain Marseille-P2769 16S ribosomal RNA, partial sequence | 335 | 100% | 1.00E-91 | 90.61% | NR_144748.1 |
|  | Deinococcus aetherius strain ST0316 16S ribosomal RNA, partial sequence | 393 | 100% | 4.00E-109 | 95.51% | NR_112189.1 |
