# Supplementary material for: Characterization of black patina from the Tiber River embankments using Next-Generation Sequencing
Source: PLoS One. 2020 Jan 9;15(1):e0227639. doi: 10.1371/journal.pone.0227639 (PMC6952188; doi:10.1371/journal.pone.0227639)
Supplement: S4 Table — (DOCX) [file pone.0227639.s005.docx]

**Table S4**

**S4 Table. Average taxonomic abundance (with standard deviation) for each fungal genus in uncolonized controls and Black patina samples.**

| **OTUs taxonomic assignment** | **Uncolonized** | **sd** | **Black patina** | **sd** |
| --- | --- | --- | --- | --- |
| Unassigned;-;-;-;-;-;- | 0.00 | 0.00 | 8.06 | 3.96 |
| k-Chromista;p-unidentified;c-unidentified;o-unidentified;f-unidentified;g-unidentified;s-unidentified | 0.00 | 0.00 | 0.03 | 0.10 |
| k-Fungi;-;-;-;-;-;- | 17.17 | 40.60 | 9.83 | 4.70 |
| Ascomycota;-;-;-;-;- | 0.00 | 0.00 | 1.33 | 1.09 |
| Ascomycota;c-Dothideomycetes;-;-;-;- | 0.00 | 0.00 | 0.01 | 0.04 |
| Ascomycota;c-Dothideomycetes;o-Botryosphaeriales;f-Botryosphaeriaceae;g-Diplodia;- | 0.00 | 0.00 | 0.01 | 0.02 |
| Ascomycota;c-Dothideomycetes;o-Capnodiales;-;-;- | 0.00 | 0.00 | 0.12 | 0.27 |
| Ascomycota;c-Dothideomycetes;o-Capnodiales;f-Cladosporiaceae;g-Cladosporium;- | 0.00 | 0.00 | 0.08 | 0.14 |
| Ascomycota;c-Dothideomycetes;o-Capnodiales;f-Extremaceae;g-Vermiconia;s-Vermiconia | 0.00 | 0.00 | 0.05 | 0.13 |
| Ascomycota;c-Dothideomycetes;o-Capnodiales;f-Mycosphaerellaceae;-;- | 0.00 | 0.00 | 0.41 | 0.39 |
| Ascomycota;c-Dothideomycetes;o-Dothideales;f-Aureobasidiaceae;g-Aureobasidium;s-Aureobasidium | 0.00 | 0.00 | 25.36 | 18.09 |
| Ascomycota;c-Dothideomycetes;o-Pleosporales;-;-;- | 0.00 | 0.00 | 1.61 | 1.11 |
| Ascomycota;c-Dothideomycetes;o-Pleosporales;f-Didymellaceae;-;- | 0.00 | 0.00 | 3.34 | 1.57 |
| Ascomycota;c-Dothideomycetes;o-Pleosporales;f-Didymellaceae;g-Ascochyta;s-Ascochyta | 0.00 | 0.00 | 0.01 | 0.02 |
| Ascomycota;c-Dothideomycetes;o-Pleosporales;f-Didymosphaeriaceae;-;- | 0.00 | 0.00 | 0.08 | 0.21 |
| Ascomycota;c-Dothideomycetes;o-Pleosporales;f-Didymosphaeriaceae;g-Paraconiothyrium;s-unidentified | 0.00 | 0.00 | 0.04 | 0.13 |
| Ascomycota;c-Dothideomycetes;o-Pleosporales;f-Lentitheciaceae;-;- | 0.00 | 0.00 | 2.54 | 0.95 |
| Ascomycota;c-Dothideomycetes;o-Pleosporales;f-Leptosphaeriaceae;g-unidentified;s-unidentified | 0.00 | 0.00 | 0.02 | 0.06 |
| Ascomycota;c-Dothideomycetes;o-Pleosporales;f-Lophiotremataceae;g-Lophiotrema;s-Lophiotrema | 0.00 | 0.00 | 0.01 | 0.02 |
| Ascomycota;c-Dothideomycetes;o-Pleosporales;f-Phaeosphaeriaceae;-;- | 0.00 | 0.00 | 3.64 | 2.10 |
| Ascomycota;c-Dothideomycetes;o-Pleosporales;f-Phaeosphaeriaceae;g-Chaetosphaeronema;s-unidentified | 0.00 | 0.00 | 0.07 | 0.18 |
| Ascomycota;c-Dothideomycetes;o-Pleosporales;f-Phaeosphaeriaceae;g-Neosetophoma;s-Neosetophoma | 0.00 | 0.00 | 0.10 | 0.17 |
| Ascomycota;c-Dothideomycetes;o-Pleosporales;f-Phaeosphaeriaceae;g-Sclerostagonospora;- | 0.00 | 0.00 | 0.07 | 0.17 |
| Ascomycota;c-Dothideomycetes;o-Pleosporales;f-Phaeosphaeriaceae;g-Sclerostagonospora;s-Sclerostagonospora | 0.00 | 0.00 | 0.12 | 0.18 |
| Ascomycota;c-Dothideomycetes;o-Pleosporales;f-Phaeosphaeriaceae;g-Setophaeosphaeria;s-Setophaeosphaeria | 0.00 | 0.00 | 1.25 | 0.84 |
| Ascomycota;c-Dothideomycetes;o-Pleosporales;f-Phaeosphaeriaceae;g-unidentified;s-unidentified | 0.00 | 0.00 | 1.15 | 1.05 |
| Ascomycota;c-Dothideomycetes;o-Pleosporales;f-Pleosporaceae;-;- | 0.00 | 0.00 | 0.02 | 0.07 |
| Ascomycota;c-Dothideomycetes;o-Pleosporales;f-Pleosporaceae;g-Alternaria;s-Alternaria | 0.00 | 0.00 | 0.72 | 0.59 |
| Ascomycota;c-Dothideomycetes;o-Pleosporales;f-Pleosporaceae;g-Stemphylium;s-Stemphylium | 0.00 | 0.00 | 0.01 | 0.02 |
| Ascomycota;c-Dothideomycetes;o-Pleosporales;f-Pleosporales_fam_Incertae_sedis;g-Macrodiplodiopsis;s-Macrodiplodiopsis | 0.00 | 0.00 | 0.05 | 0.12 |
| Ascomycota;c-Dothideomycetes;o-Pleosporales;f-Thyridariaceae;g-Parathyridaria;s-Parathyridaria | 0.00 | 0.00 | 0.03 | 0.08 |
| Ascomycota;c-Dothideomycetes;o-Pleosporales;f-unidentified;g-unidentified;s-unidentified | 0.00 | 0.00 | 0.01 | 0.04 |
| Ascomycota;c-Eurotiomycetes;o-Chaetothyriales;-;-;- | 0.00 | 0.00 | 0.01 | 0.04 |
| Ascomycota;c-Eurotiomycetes;o-Chaetothyriales;f-Herpotrichiellaceae;g-Coniosporium;s-Coniosporium | 49.50 | 54.24 | 17.05 | 14.09 |
| Ascomycota;c-Eurotiomycetes;o-Chaetothyriales;f-Trichomeriaceae;g-Knufia;s-Knufia | 0.00 | 0.00 | 0.01 | 0.02 |
| Ascomycota;c-Eurotiomycetes;o-Verrucariales;f-Verrucariaceae;-;- | 0.00 | 0.00 | 4.23 | 10.19 |
| Ascomycota;c-Lecanoromycetes;o-Lecanorales;-;-;- | 0.00 | 0.00 | 0.39 | 1.74 |
| Ascomycota;c-Lecanoromycetes;o-Lecanorales;f-Parmeliaceae;-;- | 0.00 | 0.00 | 0.06 | 0.27 |
| Ascomycota;c-Lecanoromycetes;o-Teloschistales;f-Teloschistaceae;g-Caloplaca;s-Caloplaca | 0.00 | 0.00 | 1.72 | 5.22 |
| Ascomycota;c-Leotiomycetes;o-Erysiphales;f-Erysiphaceae;g-Erysiphe;- | 0.00 | 0.00 | 0.01 | 0.02 |
| Ascomycota;c-Leotiomycetes;o-Helotiales;f-Helotiaceae;-;- | 0.00 | 0.00 | 0.53 | 0.49 |
| Ascomycota;c-Leotiomycetes;o-Helotiales;f-Helotiaceae;g-Articulospora;s-unidentified | 0.00 | 0.00 | 0.22 | 0.30 |
| Ascomycota;c-Sordariomycetes;-;-;-;- | 0.00 | 0.00 | 0.35 | 0.88 |
| Ascomycota;c-Sordariomycetes;o-Diaporthales;-;-;- | 0.00 | 0.00 | 0.01 | 0.02 |
| Ascomycota;c-Sordariomycetes;o-Diaporthales;f-Diaporthaceae;g-Diaporthe;- | 0.00 | 0.00 | 0.33 | 0.31 |
| Ascomycota;c-Sordariomycetes;o-Glomerellales;f-Glomerellaceae;g-Colletotrichum;- | 0.00 | 0.00 | 0.03 | 0.09 |
| Ascomycota;c-Sordariomycetes;o-Hypocreales;f-Nectriaceae;-;- | 0.00 | 0.00 | 0.01 | 0.02 |
| Ascomycota;c-Sordariomycetes;o-Hypocreales;f-Stachybotryaceae;g-Myrothecium;s-unidentified | 0.00 | 0.00 | 0.02 | 0.09 |
| Ascomycota;c-Sordariomycetes;o-Xylariales;f-Amphisphaeriaceae;g-Seimatosporium;s-unidentified | 0.00 | 0.00 | 0.01 | 0.04 |
| Ascomycota;c-Sordariomycetes;o-Xylariales;f-Bartaliniaceae;-;- | 0.00 | 0.00 | 0.27 | 0.28 |
| Ascomycota;c-Sordariomycetes;o-Xylariales;f-Diatrypaceae;g-unidentified;s-unidentified | 11.88 | 29.11 | 0.00 | 0.00 |
| Ascomycota;c-Sordariomycetes;o-Xylariales;f-Xylariaceae;g-Daldinia;s-Daldinia | 0.00 | 0.00 | 0.01 | 0.02 |
| Basidiomycota;-;-;-;-;- | 0.00 | 0.00 | 1.34 | 1.00 |
| Basidiomycota;c-Agaricomycetes;-;-;-;- | 0.00 | 0.00 | 0.33 | 0.48 |
| Basidiomycota;c-Agaricomycetes;o-Agaricales;f-Agaricaceae;g-Leucocoprinus;s-Leucocoprinus | 0.00 | 0.00 | 0.06 | 0.27 |
| Basidiomycota;c-Agaricomycetes;o-Agaricales;f-Psathyrellaceae;g-Coprinopsis;s-Coprinopsis | 0.00 | 0.00 | 0.24 | 0.20 |
| Basidiomycota;c-Agaricostilbomycetes;o-Agaricostilbales;f-Kondoaceae;-;- | 0.00 | 0.00 | 0.01 | 0.04 |
| Basidiomycota;c-Agaricostilbomycetes;o-Agaricostilbales;f-Kondoaceae;g-Kondoa;s-Kondoa | 0.00 | 0.00 | 0.07 | 0.13 |
| Basidiomycota;c-Cystobasidiomycetes;-;-;-;- | 0.00 | 0.00 | 0.64 | 0.42 |
| Basidiomycota;c-Cystobasidiomycetes;o-Cystobasidiomycetes_ord_Incertae_sedis;f-Buckleyzymaceae;g-Buckleyzyma;s-Buckleyzyma | 0.00 | 0.00 | 0.40 | 0.51 |
| Basidiomycota;c-Cystobasidiomycetes;o-Cystobasidiomycetes_ord_Incertae_sedis;f-Symmetrosporaceae;g-Symmetrospora;- | 0.00 | 0.00 | 0.04 | 0.16 |
| Basidiomycota;c-Cystobasidiomycetes;o-Erythrobasidiales;f-Erythrobasidiaceae;g-Erythrobasidium;s-Erythrobasidium | 0.00 | 0.00 | 0.03 | 0.08 |
| Basidiomycota;c-Exobasidiomycetes;o-Microstromatales;f-Microstromatales_fam_Incertae_sedis;g-Pseudomicrostroma;s-Pseudomicrostroma | 0.00 | 0.00 | 0.04 | 0.07 |
| Basidiomycota;c-Malasseziomycetes;o-Malasseziales;f-Malasseziaceae;g-Malassezia;s-Malassezia | 21.45 | 40.16 | 0.00 | 0.00 |
| Basidiomycota;c-Microbotryomycetes;o-Microbotryomycetes_ord_Incertae_sedis;f-Microbotryomycetes_fam_Incertae_sedis;g-Curvibasidium | 0.00 | 0.00 | 0.01 | 0.02 |
| Basidiomycota;c-Microbotryomycetes;o-Sporidiobolales;f-Sporidiobolaceae;g-Rhodosporidiobolus | 0.00 | 0.00 | 0.02 | 0.04 |
| Basidiomycota;c-Tremellomycetes;o-Filobasidiales;f-Filobasidiaceae;g-Filobasidium;s-Filobasidium | 0.00 | 0.00 | 1.94 | 1.50 |
| Basidiomycota;c-Tremellomycetes;o-Filobasidiales;f-Filobasidiaceae;g-Naganishia;s-Naganishia | 0.00 | 0.00 | 0.05 | 0.18 |
| Basidiomycota;c-Tremellomycetes;o-Tremellales;f-Bulleribasidiaceae;g-Dioszegia;- | 0.00 | 0.00 | 0.06 | 0.14 |
| Basidiomycota;c-Tremellomycetes;o-Tremellales;f-Bulleribasidiaceae;g-Dioszegia;s-Dioszegia | 0.00 | 0.00 | 0.01 | 0.02 |
| Basidiomycota;c-Tremellomycetes;o-Tremellales;f-Bulleribasidiaceae;g-Hannaella;s-Hannaella | 0.00 | 0.00 | 0.01 | 0.04 |
| Basidiomycota;c-Tremellomycetes;o-Tremellales;f-Bulleribasidiaceae;g-Vishniacozyma;s-Vishniacozyma | 0.00 | 0.00 | 0.09 | 0.13 |
| Basidiomycota;c-Tremellomycetes;o-Tremellales;f-Rhynchogastremataceae;g-Papiliotrema;s-Papiliotrema | 0.00 | 0.00 | 0.17 | 0.29 |
| Basidiomycota;c-Tremellomycetes;o-Tremellales;f-Tremellaceae;g-Tremella;- | 0.00 | 0.00 | 0.02 | 0.05 |
| Basidiomycota;c-Tremellomycetes;o-Tremellales;f-unidentified;g-unidentified;s-unidentified | 0.00 | 0.00 | 0.03 | 0.08 |
| unidentified;c-unidentified;o-unidentified;f-unidentified;g-unidentified;s-unidentified | 0.00 | 0.00 | 9.07 | 12.64 |
| k-Plantae;p-Chlorophyta;c-Trebouxiophyceae;o-Trebouxiales;f-Trebouxiaceae;g-Trebouxia;s-Trebouxia | 0.00 | 0.00 | 0.08 | 0.26 |
