# Supplementary material for: Characterization of black patina from the Tiber River embankments using Next-Generation Sequencing
Source: PLoS One. 2020 Jan 9;15(1):e0227639. doi: 10.1371/journal.pone.0227639 (PMC6952188; doi:10.1371/journal.pone.0227639)
Supplement: Attachment — Submitted filename: Point by point response Antonelli et al.pdf [file pone.0227639.s006.pdf]

Manuscript ID PONE-D-19-21352

"Tiber's embankments black patina characterization by Next-Generation Sequencing"

Point-by-point response to reviewer's comments

Reviewer # 1

We appreciated the challenging quality of the Reviewer 1's comments that prompted us to carefully revise the manuscript to clarify experimental details and results in order to increase its readability and to better support our conclusions.

Specific points

Question 1. Black patina are common features in monuments and walls around the world. They often associated with substrata where microenvironmental conditions promote the retention of water. They can be broadly classified depending on the dominant process leading to their formation. Earlier studies highlighted the chemical, pollutant-related origin of black crusts, often associated with Sulphur-laden atmosphere and the formation of gypsum layers that harden on the stone upper layers. On the other hand, on the Tropics, the occurrence of biologically-dominated black crust have been recently reported to be predominantly linked to microbial colonization by pigmented organisms synthesizing compounds such as scytonemins and mycosporine-like substances. It appears that the authors have dealt with in this study with latter type of black crusts; they should highlight this in the title.

Answer 1. We apologize if the description of black patina, and its differences with black crusts, were not clearly defined in the introduction. *Reviewer 1* raises an important point and, albeit we feel that the definition "black patina" is already a clear statement on the biological origin of the alteration, in the new MS we highlighted that black crusts and black patinas are two distinct deterioration patterns of stone artefacts. Black crusts have a chemical origin and are defined as "Kind of crust developing generally on areas protected against direct rainfall or water runoff in urban environment. Black crusts usually adhere firmly to the substrate. They are composed mainly of particles from the atmosphere, trapped into a gypsum (CaSO4.2H2O) matrix" (Vergès-Belmin V. Illustrated glossary on stone deterioration. ICOMOS. 2008). The term patina refers to several kinds of alterations, of organic and inorganic origin, tightly adhering to the substrate. As reported in the manuscript's introduction, since the 1990s the term is also used to define an aesthetic change of rock surfaces linked to the biological colonization. Black patinas are a well-known biological alteration of stone surfaces studied since the beginning of 1900 in natural environments. In the field of the conservation of Cultural Heritage several studies covered this topic in the last decades.

Question 2. *Secondly, I find that the abstract fails to display the main findings. NGS studies about subaerial communities are comparatively less studied than other terrestrial habitats, the authors should take advantage of this gap of knowledge and exploit more their data.*

Answer 2. We thank the *Reviewer 1* for this suggestion. The abstract was modified.

Question 3. *Third, the introduction could certainly be improved by adding relevant references that highlight the biological composition of black crust in stone surfaces.*

Answer 3. The biological composition of the patina is now better described in the introduction and further information were added concerning the bacteria composition.

Question 4. *I feel that the authors could improve their study by providing more details about the nature on substratum (mineralogy of stone, from bibliographic data), the prevailing microenvironment (from nearly meteorological stations) and orientation of surfaces.*
Answer 4. We agreed with the Reviewer 1 and the new materials and methods section contains the data concerning the composition of travertine and thermo-hygrometric trends registered in the city.

Question 5*. I could notice that samples were taken from either Black or White areas (replicates). A this point I am not sure the white-derived samples are originated from a "white platina" or non-colonized (at least by visual inspection) surfaces. A patina normally refers to surface alteration by a number of processes that result on modification of the upper layer, sometimes pure staining (aesthetic) but also chemical transformation of the upper layer. Please clarify this point.*
Answer 5. The Reviewer is right: The term "white" could be confusing. We collected this powder from apparently not colonized area and not from "white patina". We modified the legend of the samples changing "white" in "uncolonized" area and we added a new table with the ID of the samples and corresponding DNA extraction values (Table S1).

Question 6. *Also, please state if they were taken randomly. In addition, indicate how you managed with graffiti that is obvious on the image. Adding arrows to the sampled areas would have the reader to clearly identity the origin of samples*.
Answer 6. The materials and methods section was clarified as requested and arrows were added in the figure.

Question 7. *The apparent lack of correspondence of certain organisms not identified by NGS but seen by microscopy could be explained by the non-efficient extraction of nucleic acids, we have seen this in the past with thick-sheathed cyanobacteria.*
Answer 7. We welcomed the comment of the *Reviewer* and we agree that difficulties in lysis of specific bacterial sheaths and consequently DNA extraction could be responsible for the not complete correspondence with the microscopic analyses results. For this reason we added a specific note concerning this point in the revised manuscript.

Question 8. *Overall, I find that the results need be better contrasted with published studies based on both non-culture dependent and culture-dependent studies from epilithic habitats and highlight the main findings.*
Answer 8. The text was modified as requested.

Question 9. *Please also state and provide relevant references as to how this type of NGS-based study can provide relevant information regarding conservation issues.*
Answer 9. We would like to emphasize that this is the first study that apply NGS to the characterization of black patinas, so no references are available about the relevance of this technique in the field of conservation. The importance of the obtained data has been underlined in the text.

Question 10. I*n addition, the conclusion section needs to be enhanced to fully be supported by the results.*
Answer 10. The conclusion section was extensively revised and we think that now it's better supported by the results.

Reviewer # 2

General comment
The authors present an interesting study by analyzing the microbiota of a black patina often found over travertine embankments of Tiber river in Rome. For this reason, Next-Generation sequencing techniques, through Illumina platform, were applied in order to identify and characterize different communities of bacteria, fungi and algae, as a mean to understand the possible effects of these colonial organisms to the studied material. The study is well organized, presents relevant data, especially the statistics results and graphics, and the manuscript is well written.

We have been very happy to learn that the Reviewer 2 found the MS “an interesting study”, “well organized”, that “presents relevant data” and “well written”.

Specific points

Question 1. *Introduction. Line 48 – replace “works of art” for “artworks”*
Answer 1. The text was modified as indicated.

Question 2. *In this section a final paragraph with the study objectives is missing. Please add the objectives of the work to complete well the Introduction section.*
Answer 2. The introduction was improved and the objectives of the work were added

Question 3. *Materials and Methods. Line 103 – replace the number “1” for number “2”. In this manuscript “Materials and Methods” are the section number 2.*
Answer 3. The text was modified as indicated.

Question 4. *Results. In sub-sections “3.1 Bacterial Community” and “3.2 Fungal community” please add the percentages of abundance of the described taxa. This is relevant data in such NGS study and is missing on this section*.
Answer 4. We thank the Reviewer 2 for the suggestion**.** Two tables (one for bacteria and one for fungi) containing the percentages of abundance for each taxon (averages and standard deviations) in black patinas and uncolonized stone have been produced as supplementary inserts.

Question 5. *Discussion. I advise the authors to add an introductory paragraph to this section, instead of starting immediately with the results discussion. It would be good to start with some statement (3-4 lines) regarding the importance of the used methodology to characterize the microbiota communities of the black patina present in such important Cultural Heritage structure, which was actually the aim of this study.*
Answer 5. We agree with the *Reviewer 2* and an introductory statement was added to the paragraph.

Reviewer # 3

General comment
I believe that the manuscript by Antonelli et al. “Tiber’s embankments black patina characterization by Next-Generation Sequencing” (ref: PONE-D-19-21352) is a very interesting study concerning the complete metagenomic analysis of black patinas in an important stone monument. In my opinion, the topic is relevant and deserves to be highlighted. I also would like to pinpoint that the application of Shotgun metagenomics is currently rather scarce in the field, thus turning the article highly innovative. I recommend the acceptance of the article after the authors conduct major revisions in the manuscript, and some crucial points are addressed.

We would like to thank the *Reviewer* 3 for considering our study “highly innovative” and for his/her comments/suggestions that prompted us to improve the quality of the MS.

Specific points

Question 1. *The article should be proofread by an English native speaker.*
Answer 1. The manuscript has been proofread by an English native speaker

Question 2. *The term 18S ITS should be replaced for ITS2-rDNA sub-region, since from my understanding the 18S region (SSU) was not considered during the course of this study.*
Answer 2. The word "*18S ITS*" has been replaced with " *ITS2-rDNA* " in the revised manuscript

Question 3. *Introduction section, Lines 49-60 and Lines 75-76.*
Comment: Please consider rephrasing these sentences. They are too long and their structure could be improved.
Answer 3. The text was modified as suggested.

Question 4. *Line 71 and Line 284.*
Comment: In line 71. the reference for Pentecost (1992), in this case [45], is missing. In line 284, the reference for Albertano (2012), in this case [18], is also missing. I advise the authors to double check their references along the manuscript and in the references.
Answer 4. The manuscript was checked and all the missing references were added.

Question 5. *Introduction section, Line 64-84.*
Comment: This part of the introduction section is only focused in Phototrophic microorganisms and Fungi. I believe that the role (if any) and presence of bacteria in black patinas (if previously studied), should also be highlighted in this part.
Answer 5. We agree with the Reviewer and, although the role of the bacteria in subaerial biofilms had not been clarified jet, we added a section in the introduction regarding these microorganisms.

Question 6. *Introduction section, Line 93-95.*
Comment: The aims of the study should be clearer.
Answer 6. The introduction was modified and the aims of the study were clarified

Question 7. *Introduction section, Line 95-99.*
Comment: I believe this part should be moved to the Materials and methods- Sample collection and description sub-section.
Answer 7. The text was modified as indicated.

Question 8. *Materials and methods section, Line 103. Discussion section, Line 274.*
Comment: In line 103, this section should be: 2. Materials and methods. In the current form is displayed as 1. Materials and methods. In line 274, this section should be: 4. Discussion. In the current form lacks numbering.
Answer 8. The text was modified and the paragraph numbers were removed as requested by the Plos One author’s guidelines

Question 9. *Materials and methods section; 2.1 Sample collection and description.*
Comment: I believe that the manuscript could benefit from the addition of a table displaying the distinct samples IDs and further metadata. The table could also display which samples were able to be studied through the distinct metagenomic methodologies applied.

Answer 9. *Reviewer 3* is right. We added a table (Table S1), displaying the distinct sample IDs, specific amount of DNA extraction, corresponding obtained library and sequencing quality checkpoint.

Question 10. *Materials and methods section; 2.1 Sample collection and description.*
Comment: For the microscopical analysis, were the samples randomly selected? What were the criteria for the selection of these samples? Which samples (ID) were studied?
Answer 10. We apologize if the description of this part of the material and methods section was not clear. The section "Sample collection and description" was revised and now we believe that we address the specific concerns regarding the selection of the samples.

Question 11. *Materials and methods section; 2.2 DNA extraction, library preparation and sequencing.*
Comment: Please provide further details regarding the DNA extraction, library preparation and sequencing.
Answer 11. The section "DNA extraction" in material and methods was added with more detailed information, whereas the sections "Sample collection and description" and "Sequencing Data Analysis" were revised.

Question 12. *Results section; 3.2 Bacterial community, Lines 204-208.*
Comment: This part should be moved to the discussion section.
Answer 12. The text was modified as suggested.

Question 13. *Results (e.g. Lines 227-230), Discussion (e.g. Lines 341-383) and Figures 4 and 8.*
Comment: These parts highlight my main concern with the manuscript. In general, the application of the Illumina MiSeq methodology targeting the ITS2 rDNA sub-region does not allow a proper and accurate taxonomic annotation to the species level. I believe that these parts as well as the figures above mentioned, need to highlight a taxonomic annotation at the genus level, and therefore require to be updated. I don´t feel that the discussion bulk will be affected by this decision. However, I do acknowledge that this change will require several parts of the manuscript to be updated.
Answer 13. The figures were modified as requested and the Results and discussion sections were updated

Question 14. *Figure 6 legend needs further information, namely the distinct COG categories.*
Answer 14. The revised version of Figure 6 contains the descriptions for each COG category.

Question 15. *Discussion section.*
Comment: Given the quality of the results, some parts of the discussion section could be deeper debated. For example, the authors state that some bacteria taxa can be linked to the presence of substances of anthropogenic origin. Pollution is known to act as an ecological pressure on cyanobacterial communities. Could the presence of these cyanobacterial taxa be linked to extremotolerance profiles allowing them to thrive in these conditions? MCF are also known for their tolerance to various environmental factors, could their presence in the B samples also be explained by their high metabolic capacities?
Answer 15. The discussions were modified as requested. Two paragraphs concerning the cellular and metabolic peculiarities of cyanobacteria and MCF were added.
